# Clinical Vigilance Overcomes Imaging Limitations in Jejunal Perforation: A Case Report

**DOI:** 10.7759/cureus.79278

**Published:** 2025-02-19

**Authors:** Riyam M AL-Ajrash

**Affiliations:** 1 Department of Surgery, Al-Yarmouk Teaching Hospital, Baghdad, IRQ

**Keywords:** blunt abdominal trauma, diagnostic imaging, exploratory laparotomy, hollow viscus injury, jejunal perforation

## Abstract

Jejunal perforation following blunt abdominal trauma is a rare but critical condition that poses significant diagnostic challenges due to its subtle clinical and radiographic presentations. This case report describes a 29-year-old male who sustained blunt abdominal trauma in a motorcycle accident. Despite initial imaging, including an erect chest X-ray showing no pneumoperitoneum and CT with intravenous contrast revealing only splenic injury, the diagnosis of jejunal perforation was delayed until clinical deterioration prompted exploratory laparotomy. The findings highlight the limitations of plain radiographs and the absence of oral contrast in CT protocols for detecting hollow viscus injuries. This case underscores the importance of maintaining a high index of suspicion, employing advanced imaging techniques, and ensuring prompt surgical intervention when clinical signs indicate progression. Enhanced diagnostic strategies, including the use of both oral and IV contrast in CT imaging, are crucial to improving outcomes in similar cases.

## Introduction

Blunt abdominal trauma continues to represent one of the most serious mechanisms of injury causing morbidity and mortality; it contributes substantially to the number of injuries from both motor vehicle and high-impact-related accidents. Such a mechanism may cause variable intra-abdominal injuries such as solid organ lacerations, vascular injuries, and hollow viscus perforation. Of the available options, the rare but very challenging situations from a diagnostic and therapeutic point of view include hollow viscus perforation, such as jejunal perforation (perforating small bowel injury accounted for less than 0.3% of blunt admissions) [1]. Most of these injuries have a very subtle clinical and radiographic presentation and, if diagnosis is delayed, may result in adverse outcomes [2].

The limitations of conventional imaging modalities further compound these diagnostic challenges. For instance, erect chest X-rays are conventionally used in the detection of free air under the diaphragm as a sign of pneumoperitoneum; their sensitivity for detecting hollow viscus injuries remains very low, especially in those with small perforations or those who have not taken up oral contrast. While advanced imaging techniques, such as CT with oral and IV contrast, are more sensitive and specific, they are not always used in the initial evaluation in resource-poor settings. A literature review has emphasized the need for a high index of suspicion and a multimodal diagnostic approach to avoid delays in management [2].

It represents a case report on a young male who developed blunt abdominal trauma after a motorcycle accident. The case underlines various diagnostic dilemmas, limitations in the findings on initial imaging, and the contribution of clinical vigilance. This case is reviewed because a broad-based diagnostic approach, followed by timely surgical intervention, improves the overall results in such patients. Conclusions from previous literature also emphasize the need for early diagnosis and proper management of hollow viscus injuries [3,4].

## Case presentation

**Figure 1:**
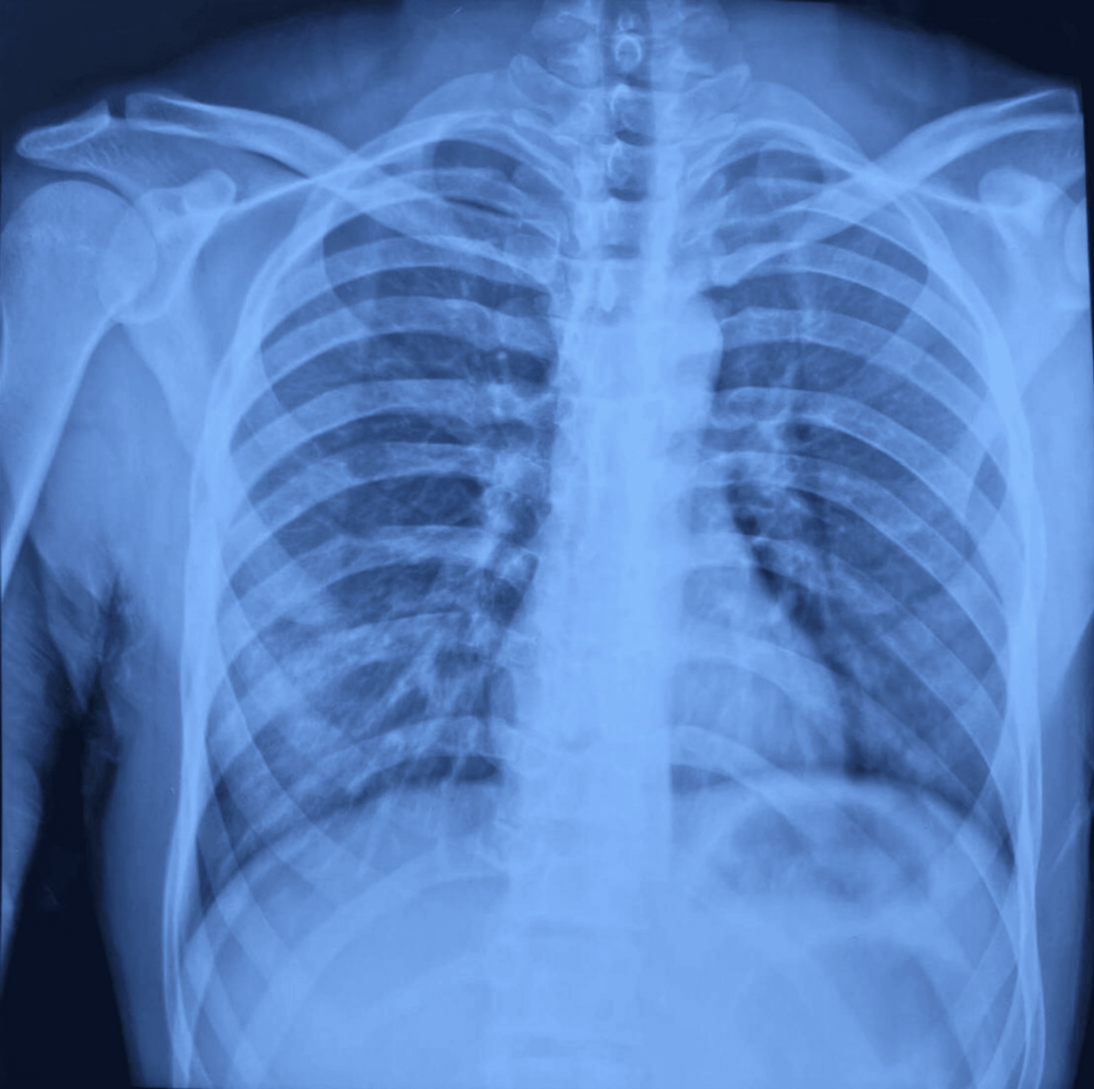
Erect chest X-ray of a 29-year-old male following blunt abdominal trauma, showing no evidence of free air under the diaphragm, effectively ruling out pneumoperitoneum at the time of the initial evaluation.

A 29-year-old male was brought to the emergency department following a motorcycle accident, which resulted in a blunt abdominal injury. On presentation, he exhibited pallor and tachycardia of 110 beats/min with a blood pressure of 90/60 mmHg. Physical examination revealed a rigid abdomen, indicative of peritonitis. A focused assessment with sonography for trauma (FAST) demonstrated a moderate amount of free fluid in the abdomen. However, an erect chest X-ray showed no air under the diaphragm, ruling out pneumoperitoneum (free air in the peritoneal cavity) at the time (Figure 1). Blood analysis showed a white blood cell (WBC) count of 13 x 10^3^/μl and hematocrit of 39.2% with a platelet count of 150 x 10^3^/μl.

**Figure 2:**
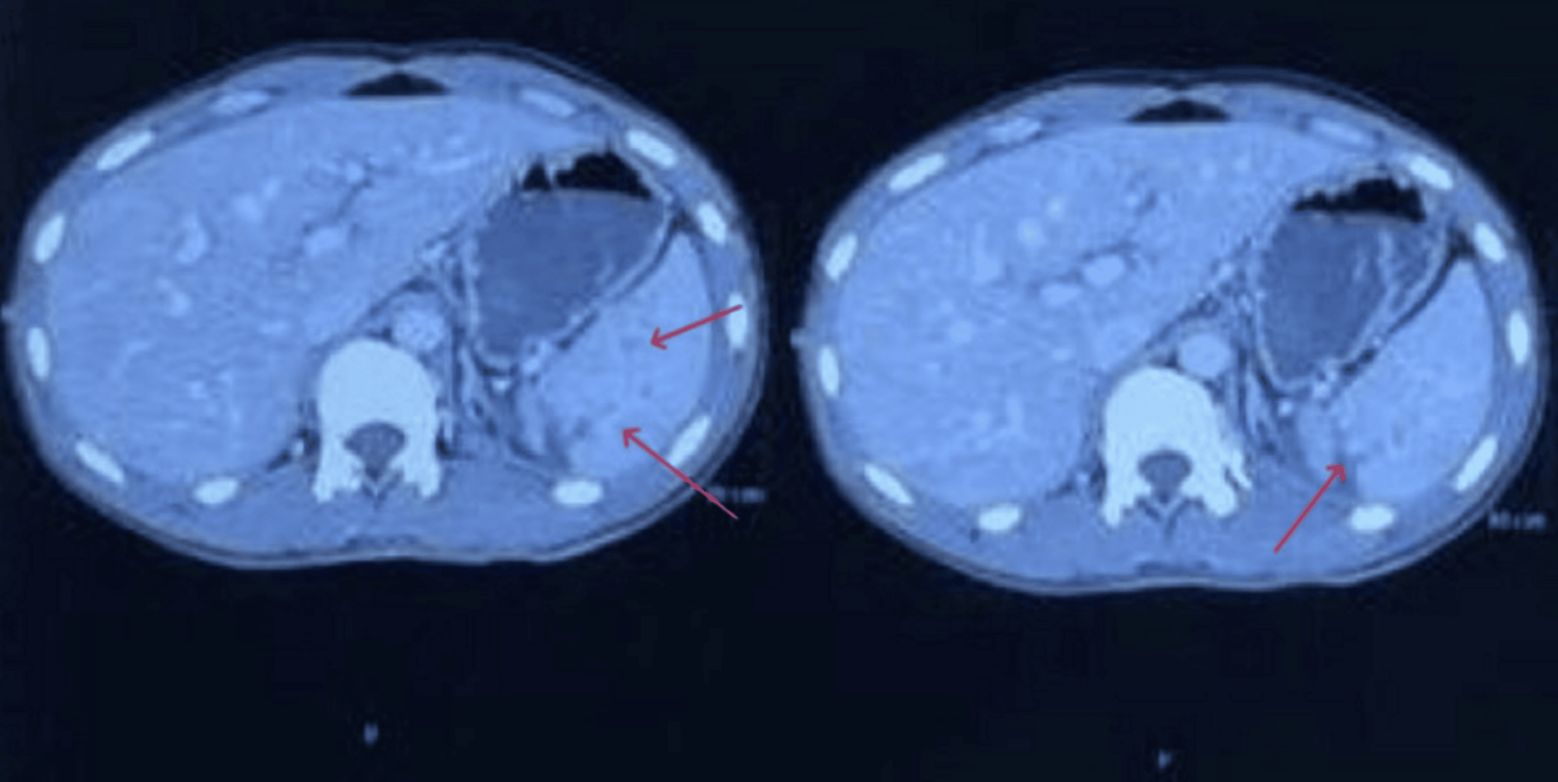
Contrast-enhanced computed tomography (CT) scans of the abdomen of a 29-year-old male following blunt abdominal trauma, demonstrating multiple lacerations within the splenic parenchyma, each measuring less than 3 cm, consistent with a grade 2 splenic injury.

This absence of free air likely contributed to a delay in identifying a hollow viscus injury. CT imaging with intravenous (IV) contrast revealed multiple lacerations within the splenic parenchyma, each measuring less than 3 cm and extending from the capsule, consistent with a grade 2 splenic injury (Figure 2). Based on these findings, the patient was managed conservatively, as no clear evidence of bowel perforation was apparent.

Despite initial stabilization, the patient’s condition worsened over the next four days. He developed severe abdominal pain, generalized peritonitis, and marked abdominal rigidity, strongly raising suspicion of a hollow viscus injury. Repeat imaging was deemed unnecessary due to the clear clinical deterioration.

Under general anesthesia with an endotracheal tube inserted, an exploratory laparotomy was performed through a midline incision, revealing significant hemoperitoneum with approximately 400 mL of blood and intestinal content contamination. Careful inspection of the abdominal cavity identified a 1 cm perforation in the jejunum, located approximately 15 cm from the duodenojejunal junction, with surrounding inflammatory changes and fibrinous exudates. The affected jejunal segment was removed with approximately 6 cm segmental resection with a hand-sewn end-to-end anastomosis using a two-layer technique (inner absorbable sutures and outer seromuscular interrupted sutures). The jejunal perforation, which was missed during the initial evaluation, underscores the limitations of relying solely on erect chest X-rays for diagnosing hollow viscus perforations, particularly when oral contrast is not utilized.

Additionally, the splenic injury had progressed to active bleeding with capsular disruption and parenchymal lacerations extending into the hilum. Hemostasis attempts with electrocautery and hemostatic agents were unsuccessful, leading to a decision for splenectomy. The splenic pedicle was carefully dissected and ligated using a combination of sutures and vascular clips, followed by complete spleen removal. Hemostasis was ensured, and a thorough peritoneal lavage with warm saline was performed to minimize the risk of intra-abdominal infection.

Two abdominal drains were placed (one in the left upper quadrant near the splenic bed and another in the pelvis) to monitor for potential postoperative bleeding or leakage from the anastomosis. The abdominal wall was closed in layers using absorbable sutures for the peritoneum and fascia, with skin closure performed using the vertical mattress technique.

The patient was transferred to the intensive care unit (ICU) for hemodynamic stabilization and close monitoring. Serial abdominal examinations were performed to assess for early signs of peritonitis or anastomotic leakage. After one day, the patient was transferred to the general surgical ward. Enteral feeding was gradually introduced starting with liquids. The patient was mobilized early to reduce the risk of postoperative ileus and pulmonary complications. To prevent overwhelming post-splenectomy infection (OPSI), vaccinations were scheduled before discharge. The patient was discharged on the seventh postoperative day. Follow-up in the hospital surgical clinic after one week for drain removal and check for wound healing and infection was made.

Long-term follow-up for the patient consisted of a multidisciplinary approach involving surgeons, gastroenterologists, and infectious disease specialists. The primary goals are to prevent infections, ensure proper digestion and absorption, and detect complications early.

## Discussion

Jejunal perforation following blunt abdominal trauma is an uncommon but serious complication, and diagnosis is challenging due to the nonspecific nature of symptoms and subtle radiographic findings. A study by Bruscagin et al. found that plain radiographs detected pneumoperitoneum in only 31% of blunt trauma patients with bowel perforations [5]. The diagnosis was delayed in this case since no pneumoperitoneum was seen on the initial erect chest X-ray, which highlights the limitations of relying on this modality in identifying hollow viscus injuries. Similar findings have been reported in other cases where pneumoperitoneum was absent on plain radiographs, which further indicates the need for alternative imaging strategies to improve diagnostic accuracy [6].

These are increased under conditions when imaging protocols do not include oral contrast. CT with both IV and oral contrast tends to increase detection rates due to the bowel injury as a source of extravasation or may reflect very slight changes that are produced by oral contrast along the wall. A study comparing triple-contrast CT (oral, rectal, and IV) to IV contrast-only CT found that the addition of oral and rectal contrast improved the accuracy, specificity, and positive predictive value in detecting bowel injuries from penetrating trauma. Notably, the presence of oral and/or rectal contrast extravasation was 100% predictive of bowel injury [7]. Therefore, the lack of oral contrast also likely contributed in this case to missing the jejunal perforation in the first review of their CT studies. This fact is corroborated by numerous studies where it was demonstrated that CT with oral and IV contrast is a better modality for the diagnosis of hollow viscus injuries, especially those without associated radiographic signs such as pneumoperitoneum [8,9].

Uniqueness in this scenario is represented by late diagnosis notwithstanding early imaging; hence, it again points out the importance of high clinical suspicions of hollow viscera injuries being continually kept in individuals with blunt abdominal trauma. Clinically progressing deteriorations that included progressive pain and generalized peritonitis in this present case should require timely surgical intervention. Other reports have also highlighted the importance of serial clinical examinations and the need for early intervention when imaging findings are indeterminate [10].

Furthermore, this case contributes to the general knowledge regarding blunt abdominal trauma by reemphasizing the limitations of conventional radiographic techniques. While plain radiographs are widely available, they have a low sensitivity for hollow viscus injuries, especially isolated jejunal perforations. Further, free air may not be present on imaging in several instances since the injury may be so minimal or associated with only a slight amount of air leakage. Advanced imaging modalities and a multimodal diagnostic approach are fundamental in such cases, as is repeating imaging or laparotomy as clinically indicated [11].

This case report derives its importance from adding to knowledge related to the understanding of diagnostic pitfalls associated with hollow viscus injuries. It restates the inclusion of oral contrast in all CT imaging protocols for suspected bowel injuries and increases clinical vigilance in cases that present with negative initial imaging. These takeaways have immediate translational applications in clinical management and the implementation of appropriate diagnostic interventions to ensure proper patient outcomes.

## Conclusions

This case represents the limitation of plain radiographs and the importance of a comprehensive diagnostic approach in blunt abdominal trauma. Clinicians should focus on CT imaging with both oral and IV contrast in suspected hollow viscus injuries and maintain a high index of suspicion when clinical deterioration occurs to ensure timely intervention and improved patient outcomes.
